# High sensitivity C-reactive protein to prealbumin ratio measurement as a marker of the prognosis in acute coronary syndrome

**DOI:** 10.1038/s41598-019-48189-y

**Published:** 2019-08-09

**Authors:** Wei Wang, Dong Ren, Chun-Song Wang, Tai Li, Heng-Chen Yao

**Affiliations:** Department of Cardiology, Liaocheng People’s Hospital Affiliated to Shandong University and Clinical School of Shandong First Medical University, Liaocheng, 252000 P.R. China

**Keywords:** Cardiology, Risk factors

## Abstract

The study aimed to determine whether high sensitivity C-reactive protein to prealbumin (hs-CRP/PAB) ratio could be used to predict in-hospital major adverse cardiac events (MACE) in patients with acute coronary syndrome (ACS). A total of 659 patients with ACS were included in the study. Patients were divided into two groups: high hs-CRP/PAB ratio group (hs-CRP/PAB ≥0.010) and low hs-CRP/PAB ratio group (hs-CRP/PAB <0.010). MACE was defined as death, cardiogenic shock, re-infarction and acute heart failure. Logistic regression was performed and the receiver operating characteristic curve (ROC) was generated to evaluate the correlation of hs-CRP/PAB ratio and MACE in patients with ACS. The occurrence rate of MACE was significantly higher in high hs-CRP/PAB ratio group when compared with that in low hs-CRP/PAB ratio group (*P* < 0.001). Multivariable analysis determined that hs-CRP/PAB ratio was an independent predictor of MACE (adjusted odds ratio: 1.276, 95% confidence interval: 1.106–1.471, *P* = 0.001). Moreover, the area under the curve value of hs-CRP/PAB ratio for predicting MACE was higher than hs-CRP and equal to PAB. High hs-CRP/PAB ratio was considered as a prognostic parameter of MACE in ACS patients, with the predictive power equal to PAB but greater than hs-CRP.

## Introduction

Cardiovascular disease is still the leading cause of mortality in the world^1^. Therefore, it is urgent to find appropriate and effective risk factors to conduct early estimation and disease prevention. Several biomarkers have been investigated for the diagnosis and risk stratification of acute coronary syndrome (ACS): cardiac troponin, creatine kinase-myocardial band and high sensitive C-reactive protein (hs-CRP). The newly discovered nardilysin is a promising biomarker for the early diagnosis of ACS^2,3^.

Inflammation plays a vital role in the process of atherosclerosis. As a marker of inflammation, hs-CRP is synthesized by the hepatocytes and mainly regulated by interleukin-6 (IL-6)^4,5^. Accumulating evidence suggests that hs-CRP is a useful prognostic indicator for major adverse cardiovascular events (MACE) in patients with heart disease^6–8^. Prealbumin (PAB) is synthesized by the liver too and suppressed in the inflammatory environment^9,10^. Investigations have shown that PAB in both the acute and chronic heart failure patients could predict mortality^11–13^. A recent study demonstrated that the PAB level is inversely related to angiographic severity score in ACS patients^14^. In our previous study, we found that PAB could independently predict MACE in patients with ACS^15^.

Both hs-CRP and PAB are produced by hepatocytes and can be measured in the blood. The newly introduced hs-CRP/PAB ratio reflects the patient’s inflammatory status better than either hs-CRP or PAB alone. Investigations have demonstrated that hs-CRP/PAB ratio is superior to either hs-CRP or PAB alone in assessing not only the severity but the prognosis of patients with acute kidney injury, fistula closure and other critically patients^16–19^. However, up until now, there has been little research focusing on hs-CRP/PAB ratio for the prediction of clinical outcomes in ACS patients. Thus, the purpose of this research was to explore the possibility of using hs-CRP/PAB ratio to predict in-hospital MACE in patients with ACS.

## Results

### Baseline characteristics of patients

A total of 659 patients were enrolled in this study with mean age of 66.5 years and 360 patients (54.6%) were male. The demographic and clinical characteristics of the two groups are shown in Table 1. The high hs-CRP/PAB ratio group had higher hs-CRP levels (median, 0.78; quartile deviation, 1.40 mg/dL) and lower PAB levels (15.73 ± 4.39 mg/dL), with a ratio value (median, 0.054; quartile deviation, 0.101). The low hs-CRP/PAB ratio group had lower hs-CRP levels (median, 0.08; quartile deviation, 0.06 mg/dL) and higher PAB levels (19.84 ± 3.64 mg/dL), with a ratio value (median, 0.004; quartile deviation, 0.003). The high hs-CRP/PAB ratio group tended to be older in age with faster heart rates. The high hs-CRP/PAB ratio group had a higher proportion of diabetics and patients with known coronary artery disease (CAD). 333 patients underwent coronary angiography with the percentage of patients with 3-vessel disease in high hs-CRP/PAB ratio group was greater than in low hs-CRP/PAB ratio (*P* < 0.001).

**Table 1:** The characteristics of patients according to serum hs-CRP/PAB ratio level.

Characteristics	Low hs-CRP/PAB group	High hs-CRP/PAB group	*P* value
	n = 329	n = 330	
Age (year)	65.16 ± 10.37	67.80 ± 11.77	0.002
Male gender, n (%)	191 (58.1)	169 (51.2)	0.078
Current smoker, n (%)	89 (27.05)	100 (30.30)	0.356
Hypertension, n (%)	196 (59.57)	217 (65.76)	0.101
Diabetes mellitus, n (%)	78 (23.71)	102 (30.90)	0.038
Previous CAD, n (%)	36 (10.94)	59 (17.88)	0.011
Killip class II-IV, n (%)	18 (5.47)	31 (9.39)	0.055
Heart rate (bpm)	72 (14.00)	76 (22.25)	<0.001
Laboratory measurement			
Hemoglobin (g/dL)	13.60 ± 1.64	12.82 ± 1.77	<0.001
Platelet (×10^3^/μL)	224.63 ± 55.63	226.02 ± 70.17	0.778
Leukocyte (×10^3^/μL)	6.21 (1.91)	7.01 (3.35)	<0.001
Glucose (mg/dL)	95.84 (26.93)	108.18 (49.14)	<0.001
eGFR (mL/min/1.73 m^2^)	84.48 ± 27.60	82.40 ± 32.44	0.010
PAB (mg/dL)	19.84 ± 3.64	15.73 ± 4.39	<0.001
Hs-CRP/PAB ratio	0.004 (0.003)	0.054 (0.101)	<0.001
GOT (u/L)	21.00 (10.00)	24.00 (20.00)	<0.001
GPT (u/L)	19.00 (13.00)	21.00 (19.00)	0.010
LDL-cholesterol (mg/dL)	101.87 ± 34.75	101.92 ± 32.69	0.984
Hs-CRP (mg/dL)	0.08 (0.06)	0.78 (1.40)	<0.001
Angiography, n (%)	169 (51.37)	164 (49.70)	0.668
1 vessel disease	110 (33.43)	85 (25.76)	
2 vessel diseases	49 (14.89)	45 (13.64)	
3 vessel diseases	10 (3.04)	34 (10.30)	<0.001
Treatment, n (%)			
Primary PCI	12 (3.65)	17 (5.15)	0.347
LMWH	100 (30.40)	116 (35.15)	0.193
Anti-platelet	321 (97.57)	323 (97.88)	0.789
Beta-blocker	207 (62.92)	195 (59.09)	0.191
ACEI/ARB	140 (42.55)	169 (51.21)	0.026
Statin	156 (47.42)	148 (44.85)	0.508

Abbreviation: Hs-CRP: high sensitivity C-reactive protein; PAB: prealbumin; CAD: coronary artery disease; eGFR: estimated glomerular filtration rate; GOT: glutamate oxaloacetate transaminase; GPT: glutamate pyruvate transaminase; LDL: low density lipoproteins; PCI: percutaneous coronary intervention; LMWH: low molecular weight heparin; ACEI: angiotensin-converting-enzyme inhibitor; ARB: angiotensin receptor blockers.

### Comparison of MACE rate of two groups

The length of in-hospital MACE occurrence ranged from 1 to 17 days, the mean time was 7 days. The occurrence rate of in-hospital MACE was 10.62% (70 of 659 patients). The proportion of total MACE and acute heart failure was higher in the high hs-CRP/PAB ratio group as compared to the low hs-CRP/PAB ratio group (*P* < 0.001, respectively). However, there was no significant difference between the two groups in the death rate (as showed in Table 2).

**Table 2:** MACE between patients with low and high ratio of hs-CRP/PAB.

In-hospital MACE	Low hs-CRP/PAB group	High hs-CRP/PAB group	*P* value
	n = 329	n = 330	
Death, n (%)	0 (0)	5 (1.52)	0.073
Acute heart failure, n (%)	4 (1.22)	44 (13.33)	<0.001
Shock cardiogenic, n (%)	1 (0.30)	1 (0.30)	0.998
Reinfarction, n (%)	2 (0.61)	13 (3.94)	0.004
Total MACE, n (%)	7 (2.13)	63 (19.09)	<0.001

Abbreviation: MACE: major adverse cardiac events; hs-CRP: high sensitivity C-reactive protein; PAB: prealbumin.

### The results of logistic regression

In univariate analysis, high hs-CRP/PAB ratio was associated with MACE (odds ratio [OR]: 1.387, 95% CI: 1.026–1.594, *P* < 0.001). In multivariable logistic regression analysis, adjusted for age, hypertension, previous CAD, glucose, estimated glomerular filtration rate and low-density lipoproteins (LDL) cholesterol, the results showed that hs-CRP/PAB ratio remained independent and predicted MACE (adjusted OR: 1.276, 95% CI: 1.106–1.471, *P* = 0.001), as well as hs-CRP and PAB (Table 3).

**Table 3:** Univariate and multivariable logistic analysis for predictors of in-Hospital MACE.

Variables	OR	95% CI	*P* value
Univariate analysis			
Hs-CRP/PAB ratio	1.387	1.026–1.594	<0.001
Hs-CRP	1.316	1.144–1.514	<0.001
PAB	0.802	0.758–0.849	<0.001
Age	1.041	1.016–1.066	0.001
Male gender	1.197	0.724–1.978	0.484
Current smoker	1.158	0.678–1.979	0.591
Previous CAD	5.194	3.022–8.927	<0.001
Hypertension	1.245	0.736–2.106	0.414
Diabetes mellitus	1.448	0.855–2.451	0.168
eGFR	0.985	0.976–0.994	0.001
Glucose	1.007	1.004–1.011	<0.001
Platelet	0.996	0.992–1.000	0.073
LDL-cholesterol	0.993	0.985–1.001	0.086
Anti-platelet	0.767	0.170–3.473	0.731
Multivariable analysis			
Hs-CRP/PAB ratio: model 1	1.276	1.106–1.471	0.001
Hs-CRP: model 2	1.210	1.038–1.410	0.015
PAB3: model 3	0.806	0.755–0.860	<0.001

Model 1, 2, 3 adjusted for age, eGFR, hypertension, previous CAD, glucose and LDL-cholesterol, respectively. Abbreviation: MACE: major adverse cardiac events; OR: odds ratio; CI: confidence interval; Hs-CRP: high sensitivity C-reactive protein; PAB: prealbumin; CAD: coronary artery disease; eGFR: estimated glomerular filtration rate; LDL: low density lipoproteins.

### ROC curve analysis

**Figure 1:**
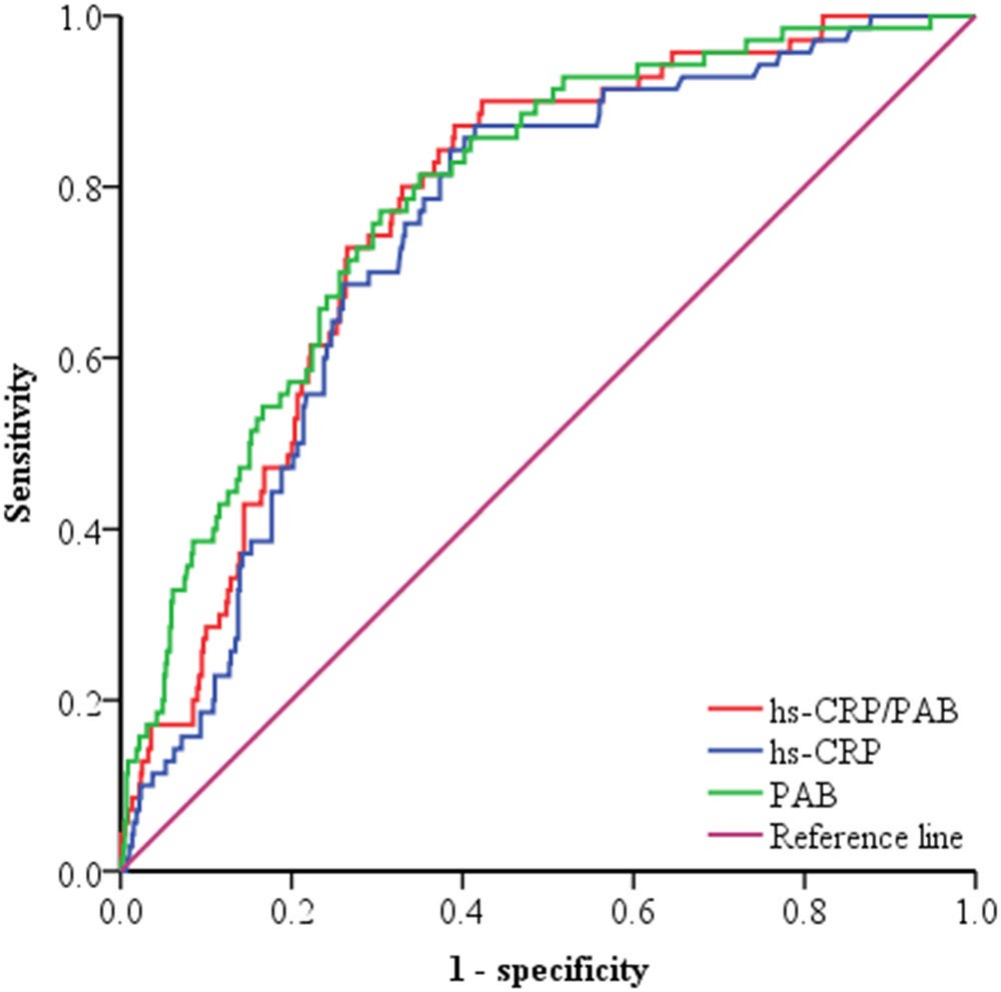
Receiver operating characteristics curves of hs-CRP, PAB and hs-CRP/PAB ratio in predicting in-hospital adverse cardiac events. The area under the curve of the hs-CRP, PAB and hs-CRP/PAB ratio is 0.743 (95% CI: 0.689–0.797), 0.787 (95% CI: 0.735–0.839) and 0.770 (95% CI: 0.719–0.820), respectively (p < 0.001). hs-CRP: high sensitivity C-reactive protein; PAB: prealbumin.

To further investigate the value of hs-CRP/PAB ratio, hs-CRP and PAB in predicting MACE, we performed receiver operating characteristic (ROC) curves (Fig. 1). The best cut-off value of the hs-CRP/PAB ratio is 0.011 with a sensitivity of 90.0% and a specificity of 57.7%, which was close to the median value of hs-CRP/PAB ratio (0.010). Both hs-CRP and PAB correlated with hs-CRP/PAB ratio (Spearman’s r: 0.975 and -0.551, respectively, both *P* < 0.001). The area under the curve (AUC) value of hs-CRP/PAB ratio was significantly higher than hs-CRP (0.770; 95% CI: 0.719–0.820 vs 0.743; 95% CI: 0.689–0.797) (Z = 2.020, *P* < 0.05), while no statistically significant difference was found between hs-CRP/PAB ratio and PAB (0.770; 95% CI: 0.719–0.820 vs 0.787; 95% CI: 0.735–0.839) (Z = 0.612, *P* > 0.05). The best cut-off value, sensitivity and specificity of hs-CRP, PAB and hs-CRP/PAB ratio for predicting MACE is shown in Table 4.

**Table 4:** Best cut-off value, sensitivity and specificity of PAB, hs-CRP and hs-CRP/PAB ratio for prediction in-Hospital of MACE.

Variables	Cut-off	Sensitivity	Specificity
PAB (mg/dL)	≤16.794	0.771	0.696
Hs-CRP (mg/dL)	>0.253	0.843	0.615
Hs-CRP/ PAB ratio	>0.011	0.900	0.577

Abbreviation: PAB: prealbumin; hs-CRP: high sensitivity C-reactive protein.

## Discussion

This study was a proof-of-concept study which served to demonstrate the hypothesis that the ratio of hs-CRP/PAB could be used as an early biomarker, to predict the in-hospital MACE in patients with ACS. In the present study the most interesting finding was the incidence rate of MACE was increased in the high hs-CRP/PAB ratio group. After adjusting for other confounding factors, the association between hs-CRP/PAB ratio and poor outcomes was still evident. Thus, we believe that hs-CRP/PAB ratio could be very helpful to predict MACE in patients with ACS.

Many biomarkers were associated with the development and progression of coronary heart disease^2,3^. In the past, the role of hs-CRP in cardiovascular disease was controversial as a risk marker. Recent studies showed that hs-CRP plays an important role in the progression of atherothrombosis. Hs-CRP elevation is a marker of inflammation that can induce vascular remodeling and coronary atherosclerotic plaque rupture^20,21^. Elevation of hs-CRP levels were also observed and associated with adipocytokine imbalance^22^, which result in visceral fat accumulation and thereby increase the risk of ACS development. Moreover, elevated hs-CRP levels were associated with reduced abilities in clearance of oxidative stressors and inflammatory mediators, thus increase the incidence of cardiovascular events^23^. It has been reported that elevated hs-CRP levels were associated with increased risk of adverse cardiovascular outcomes in patients with different CAD phenotypes^24–26^. Aguilar *et al*. focused on stable CAD and enrolled 3319 patients and followed up for 37 months, they found that increased levels of hs-CRP were associated with higher risk of adverse cardiac events^24^. The FRISC study included 917 patients with unstable coronary artery disease and the investigators found that elevated CRP levels were strongly associated with the long-term risk of death from heart disease^25^. In the current interventional era, higher hs-CRP levels at admission were associated with lower reperfusion success^27^. In accordance with these results, we have observed that hs-CRP could predict in-hospital MACE in patients with ACS. Furthermore, elevated hs-CRP levels were associated with the severity of CAD^26^; similarly, in our study, we also found patients with 3-vessel disease tended to have higher levels of hs-CRP.

However, using of hs-CRP alone to predict MACE in patients with ACS may be limited. Inflammation can induce malnutrition, which may exert a negative effect on the management of inflammation^18^. PAB as a parameter in the evaluation of nourishment state was suppressed in an inflammatory environment^10^. Observations show that PAB is transferred by high-density lipoproteins (HDL); the absence of PAB can affect the stability of the HDL particles, and then reduce its protective effect on the heart^28^. Low levels of PAB in malnourished patients may parallel with vitamin C deficiency^29^, which may link with adverse cardiac events, since vitamin C plays a key role in antioxidant and anti-inflammatory process^30^. Decreased levels of PAB could increase free thyroxine, which was associated with adverse outcomes in patients with acute myocardial infarction^31^. Clinical studies have revealed that a lower level of serum PAB was associated with prognosis of heart failure^11–13^, higher overall mortality in critically ill patients^32^, and negatively associated with angiographic severity in patients with ACS^14^. The above research results illustrated that patients with low PAB levels have poor clinical outcomes.

Hs-CRP/PAB ratio reveals the balance between hs-CRP and PAB in the body and also assesses inflammatory and nutritional status of a patient’s condition. When the value of either marker changes, the ratio could change accordingly but presented a higher sensitivity than using either marker alone. To the best of our knowledge, the current study is the first time to demonstrate that hs-CRP/PAB ratio is an independent predictor of in-hospital MACE in patients with ACS. Furthermore, the prognostic value of the hs-CRP/PAB ratio for predicting in-hospital MACE is significantly greater than the traditional risk factor, which is hs-CRP. In the present study, the predictive power of hs-CRP/PAB ratio was equal to PAB, further study with more patients and longer follow-up duration may help to evaluate if hs-CRP/PAB ratio is a better index than PAB.

This is an observational study; notwithstanding, it offers a rapidly assessable marker for clinical practice. The detection of serum hs-CRP and PAB is quite convenient in recent years, the ratio of hs-CRP to PAB may aid the risk stratification of patients with ACS and have been widely available to clinicians.

The present study has several limitations. First, deaths were defined as all-cause deaths in this study and cardiovascular disease-related mortality should be further analyzed. Second, this was a single center study and a relatively small sample was recruited. Third, stable angina pectoris or asymptomatic coronary atherosclerosis patients were not recruited in the study. Fourth, the prognostic and predictive ability of hs-CRP/PAB was only investigated about the in-hospital MACE. So, larger sample size, including patients with stable angina pectoris or asymptomatic coronary atherosclerosis, and multicenter studies are needed to further confirm the initial findings of this study.

In conclusion, this study indicates that elevated hs-CRP/PAB ratio is associated with in-hospital MACE in patients with ACS and this ratio may be used as a biomarker, to predict adverse events in patients with ACS with a predictive power equal to PAB but greater than hs-CRP.

## Methods

### Study population

This cohort study was conducted from March to October 2017, ACS patients consecutively admitted to the Department of Cardiology of Liaocheng People’s Hospital, affiliated with Shandong University were enrolled in this study. Diagnosis and determination of ACS were applied according to AHA or ACCF guidelines^33,34^. The inclusion criteria were ACS patients with angina occurred in the 24 hours and agreed to participate in this study. The exclusion criteria as follows: chronic heart failure (NYHA class >II), stage V chronic kidney disease or patients on regular dialysis, hepatic cirrhosis, malignancy or valvular heart disease, concomitant acute infection, stroke or venous thromboembolism. All the protocol was approved by the Ethics Committee of Liaocheng People’s Hospital and informed consents were obtained from all participants. All research was performed in accordance with the relevant guidelines/regulations.

### Data collection and laboratory examination

After enrollment, venous blood samples were drawn immediately on admission before reperfusion therapy. Clinical presentations were assessed, and characteristic data was collected by the attending physician on admission. Cardiovascular risk factors, including age, gender, diabetes mellitus, hypertension, smoking status and previous CAD were all noted. Hypertension was defined as the use of antihypertensive drugs or blood pressure ≥140/90 mmHg. Diabetes was defined as fasting plasma glucose of at least 7.0 mmol/L or glucose level ≥11.1 mmol/L in 2-hour glucose from an oral glucose tolerance test. Coronary angiography was performed through the femoral or radial approach and the results were interpreted by two experienced interventional cardiologists. Coronary artery with diameter obstruction >50% were considered to exhibit stenosis. Blood biochemical indexes including LDL cholesterol, liver and kidney function and blood glucose were routinely performed at the hospital’s central laboratory using Hitachi 7600–120 analyzer (Hitachi High-Technologies, Tokyo, Japan). Hs-CRP and PAB were measured in the clinical laboratory, using a biochemical analyzer (Beckman Coulter AU5800, USA), according to the manufacturer’s instructions. They were analyzed by immunoturbidimetry using original reagent which supplied by Beckman Coulter.

### In-hospital MACE definitions

The endpoint of this study was in-hospital MACE, which included death, cardiogenic shock, re-infarction and acute heart failure during hospitalization. Deaths were defined as all-cause deaths. Cardiogenic shock was defined as a persistent systolic blood pressure less than 90 mm Hg, or signs and clinical manifestations of low perfusion with administration of vasopressor agents subsequently. Re-infarction was diagnosed by recurrent or newly occurring chest pain, with new ST segment changes or cardiac troponin I levels that rose again. Acute heart failure was diagnosed according to clinical signs and symptoms: such as pulmonary edema on physical examination or breathlessness symptoms, subsequent use of intravenous diuretics or positive inotropic agents. The MACE was determined by attending cardiologists who provided the treatment accordingly.

### Statistical analysis

SPSS software version 17.0 was used for statistical analysis. The median value of hs-CRP/PAB ratio (0.010) was set as the cut-off point, the patients were divided into the hs-CRP/PAB ratio ≥0.010 (high hs-CRP/PAB ratio) group and the hs-CRP/PAB ratio <0.010 (low hs-CRP/PAB ratio) group for analysis. The Kolmogorov-Smirnov test was used to assess whether continuous data were normally distributed. Student’s *t*-test was used for comparison of normally distributed continuous variables and the Mann-Whitney U test was used for comparison of values that do not normal distribution. Continuous variables were presented as mean ± standard deviation for normal distribution and median (quartile deviation) for those with non-normal distribution. A chi-square test and Fisher exact test was used to compare categorical data. The variables that may associate with in-hospital MACE were calculated in univariate analysis, those showed a univariate relationship with MACE or clinically relevant were included in the multivariable logistic regression model. Given the number of MACE available, the included variables were carefully chosen, to ensure the final model robust. ROC analysis was carried out to establish the best cut-off value of hs-CRP, PAB and hs-CRP/PAB ratio for predicting in-hospital MACE. The Z-test was used to compare the AUC values of hs-CRP, PAB and hs-CRP/PAB ratio. A *P* value < 0.05 was determined as statistically significant.

## Data Availability

The datasets generated during and/or analyzed during the current study are available from the corresponding author on reasonable request.
